# IL-6 and other biomarkers as predictors of severity in COVID-19

**DOI:** 10.1080/07853890.2020.1840621

**Published:** 2021-03-04

**Authors:** N. Broman, K. Rantasärkkä, T. Feuth, M. Valtonen, M. Waris, U. Hohenthal, E. Rintala, A. Karlsson, H. Marttila, V. Peltola, T. Vuorinen, J. Oksi

**Affiliations:** a Department of Infectious Diseases, Turku University Hospital, Turku, Finland; b Department of Clinical Microbiology, Turku University Hospital and Institute of Biomedicine, University of Turku, Turku, Finland; c Department of Pulmonary Diseases, Turku University Hospital and Department of Pulmonary Diseases and Clinical Allergology, University of Turku, Turku, Finland; d Department of Anaesthesia and Intensive Care, Turku University Hospital, Turku, Finland; e Department of Infectious Diseases, Turku University Hospital and University of Turku, Turku, Finland; f Department of Hospital Hygiene and Infection Control, Turku University Hospital, Turku, Finland; g Auria Biobank, Turku University Hospital and University of Turku, Turku, Finland; h Department of Paediatrics and Adolescent Medicine, Turku University Hospital and University of Turku, Turku, Finland

**Keywords:** COVID-19, interleukin, interferon, inflammatory phase, tocilizumab, cytokine storm, cytokine release syndrome

## Abstract

**Objective:**

Cytokine release syndrome is suggested to be the most important mechanism triggering acute respiratory distress syndrome and end organ damage in COVID-19. The severity of disease may be measured by different biomarkers.

**Methods:**

We studied markers of inflammation and coagulation as recorded in 29 patients on admission to the hospital in order to identify markers of severe COVID-19 and need of ICU.

**Results:**

Patients who were eventually admitted to ICU displayed significantly higher serum levels of interleukin-6 (IL-6), C-reactive protein (CRP), and procalcitonin. No statistical differences were found between the groups in median levels of lymphocytes, D-dimer or ferritin.

**Conclusions:**

IL-6 and CRP were the strongest predictors of severity in hospitalized patients with COVID-19.

## Introduction

A large number of trials have been registered to investigate the various candidates of immunomodulatory therapeutics for COVID-19 including tocilizumab, an IL-6 receptor antagonist, and anakinra, an IL-1 inhibitor. Both IL-1 and IL-6 are known to have a central role in development of cytokine release syndrome (CRS) in the later phase of the disease.

A recent publication suggested that blockage of interleukin-1 with anakinra in COVID-19 patients with hyperinflammation improves survival. For that study, C-reactive protein (CRP) and ferritin were used as markers of hyperinflammation to select patients that may benefit from anakinra [1].

Severity and mortality of COVID-19 are associated with coagulopathy [2] and imbalanced immune response with marked increase of interleukins IL-1 and IL-6 as well as other cytokines and eventually organ failure [3,4].

## Brief report

We studied a number of markers of inflammation and coagulation as recorded on admission in all COVID-19 patients (*n* = 29) admitted to Turku University Hospital, Finland, up to 24 May 2020, in order to identify markers of severe COVID-19 and need for ICU. Patients were divided into three groups: Group 1 included patients without ICU restrictions but who could be treated outside ICU (13/29, 45%); Group 2 included all patients who were eventually admitted to ICU (8/29, 28%); and Group 3 included all patients with ICU-restrictions based on high age and severe comorbidity, and poor prognosis of survival (8/29, 28%).

Biomarkers were taken upon admission or within the first few days. For each biomarker, only the first measurement was used for this study. The peripheral blood lymphocyte count, ferritin, CRP, procalcitonin (PCT), and D-dimer were all analysed according to standard methods. Human myxovirus resistance protein A (MxA), a cytoplasmic GTPase with direct antiviral effect and exclusively induced by type I and III interferons (IFNs), was used as a key biomarker for identifying virus infection [5,6]. Under virus invasion, MxA forms oligomer rings around virus nucleocapsid structures blocking their translation through aggregation, disruption, or prevention of translocation. MxA is detectable in peripheral blood mononuclear cells within a few hours of IFN stimulation and has a half-life of about 2.3 days, providing a specific indication of acute or very recent virus infection. On the other hand, viruses have evasion mechanisms which delay the induction or action of IFNs.

Sars-CoV-2 qRT-PCR was performed in nasopharyngeal swabs using WHO recommended primers and probe for E gene [7], whole blood samples were tested for MxA as previously described [6], and serum IL-6 levels were assayed using the BioVendor Human IL-6 Elisa kit (BioVendor, Czech Republic). IL-6 was sampled median 12.5 days after onset of symptoms and 2 days after admission to the hospital, with no significant difference among the groups.

The median age of the patients was 55 years (range 15–82 years) and 14/29 were female (48%). Body-mass index (BMI) was available in 28 patients, of them, 11 (39%) were obese. Native oxygen saturation was registered in all cases before starting supplemental oxygen. Median native oxygen saturation on presentation was 95% in the non-ICU group and 88% in the group of patients who were eventually admitted to ICU. We found an inversed correlation between native oxygen saturation on admission and IL-6 (Spearman R −0.41, *p* = .0242).

In patients eventually admitted to ICU, obesity (BMI: >30 kg/m^2^) was present in 50% of cases and BMI >35 kg/m^2^ in 25%. In these patients, the mean simplified acute physiology score II was 35, and these patients were seriously hypoxemic with 84% mean blood oxygen saturation and 6.7 kPa arterial oxygen partial pressure. Invasive ventilation was needed in 63% of patients with a mean duration of 20 days. The mean length of the ICU stay was 17 days. Three patients (38%) needed repeatedly prone position. As of for 31 May, 3/29 patients died (10%). Of them, two died during admission in our hospital and one after referral for palliative care to a local health centre. All patients treated in ICU survived – with one of them still hospitalized with a home ventilator.

In total, six patients received corticosteroids during admission. In four cases, corticosteroids were already started before diagnosis of COVID-19. Of those, corticosteroid treatment was started for asthma exacerbation in three cases and one case received low dose (5 mg) of prednisolone as maintenance therapy for polymyalgia rheumatica. In the other two cases, systemic corticosteroids were started in ICU. Four patients receiving corticosteroids were treated in ICU, in the other two patients ICU-restrictions were set. None of the non-ICU-patients received systemic corticosteroids.

**Figure 1.**
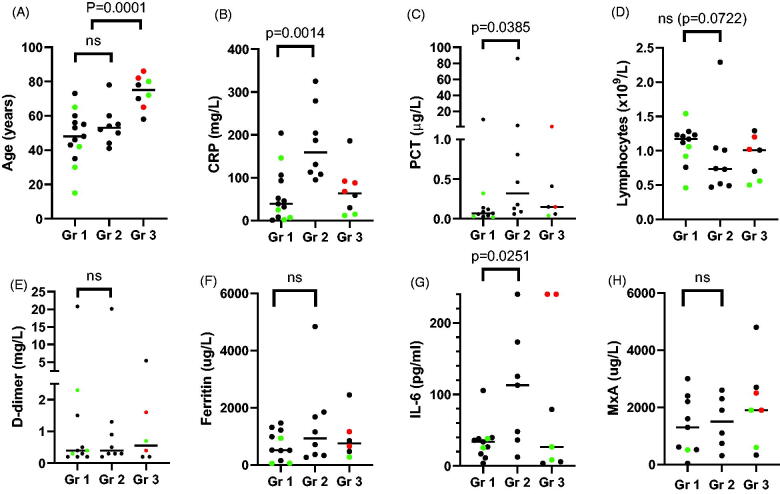
Biomarkers associated with severe COVID-19 requiring ICU-admission. All 29 patients admitted to Turku University Hospital before May 24th 2020 were included and divided into three groups. Group 1 includes all hospitalized patients without intensive care restrictions who did not require intensive care unit (ICU)-admission, Group 2 included all patients who were eventually admitted to the ICU and Group 3 includes all patients with ICU-restrictions based on age and comorbidity. Horizontal lines depict median values. Patients who eventually died are marked red, and those who did not need supplementary oxygen or other respiratory support are marked green. Differences between groups were tested for statistical significance with Mann–Whitney test. A: there was no significant difference in age between Group 1 and Group 2 (medians 48 years versus 53 years, *p*=.4886). As can be expected, age of patients with ICU-restrictions (Group 3) was significantly higher than those without restrictions (medians 75 years versus 52 years, *p*=.0001). B, C and G: Admission to ICU was associated with higher levels of CRP (medians 39 mg/L in Group 1 and 159 mg/L in Group 2, *p*=.0014), PCT (0.07 μg/L in Group 1 versus 0.32 μg/L in Group 2, *p*=.0385) and IL-6 (33.8 pg/mL in Group 1 versus 112.8 pg/mL in Group 2, *p*=.0251). D, E, F: No statistical differences were observed in level of peripheral blood lymphocytes, and serum levels of D-dimer, Ferritin. H: MxA was variably elevated in all Covid-19 patients. CRP: C-reactive protein (normal <10 mg/L); PCT: procalcitonin (normal <0.05 μg/L); Lymphocytes (normal 1.3-3.6 x10E9/L); D-dimer (normal <0.5 mg/L); P-Ferritin (normal men 30–400 μg/L, women 13–150 μg/L); S-IL-6: Interleukin-6 (normal <5.9 pg/mL); MxA: Myxovirus resistance protein A (normal <100 μg/L).

Patients who were eventually admitted to ICU displayed higher serum levels of IL-6, CRP, and PCT. The MxA levels were clearly elevated (>200 µg/L) across all groups without statistically significant difference. No statistical differences were found between the groups in median levels of lymphocytes, D-dimer or ferritin. These data are displayed in Figure 1.

In our small material, ICU admission is correlated with significantly higher IL-6 levels as compared to no need of ICU. Our findings are well in line with similar studies [8,9]. In addition, serum level of IL-6 was measured in two of three patients that eventually died and was on the upper limit of quantification (>240 pg/mL) in both of them. Another predictive biomarker for severity of the disease and need of ICU admission in our patients was CRP confirming the results of several earlier findings [10]. Blood MxA levels were variably elevated at 1–9 days after admission (median 2 days) to hospital, indicating that the patients had strong type I/III IFN response and may not, at their advanced stage of disease, have benefitted from IFN as a potential therapeutic. D-dimer has its place as a coagulation marker but at least in our material it did not predict the severity of the disease. Neither was ferritin associated to the severity of the disease. Therefore, unlike Cavalli et al., we do not support the use of ferritin in order to identify patient illegible for treatment with interleukin blockade. We consider IL-6 measurement to be a useful biomarker in clinical care of COVID-19 patients.
